# Laryngopharyngeal reflux as a potential cause of persistent local neck symptoms after total thyroidectomy

**DOI:** 10.1007/s00405-020-06223-0

**Published:** 2020-07-31

**Authors:** Calogero Cipolla, Ina Macaione, Salvatore Vieni, Mario Latteri, Angela Vullo, Giuseppa Graceffa, Eugenio Fiorentino

**Affiliations:** 1 Department of Surgical Oncological and Oral Sciences, University of Palermo, Via del Vespro 129, 90127 Palermo, Italy; 2 Istituto Zooprofilattico Sperimentale della Sicilia, Via G. Marinuzzi 3, 90129 Palermo, Italy

**Keywords:** Local neck symptoms, Thyroidectomy, Nodular goiter, Laryngopharyngeal reflux

## Abstract

**Purpose:**

Local neck symptoms (LNS) may be related to goiter, but are also reported by patients suffering from laryngeal–pharyngeal reflux (LPR). The aim of this study was to investigate whether LPR could play a role in the persistence of some LNS after total thyroidectomy (TT).

**Methods:**

A consecutive case series of 160 patients with multinodular goiter (MNG) candidate for TT were included in this study. Each patient was closely studied for both the thyroid pathology and reflux disease before and 6 months after surgery to assess the persistence of LNS after surgery.

**Results:**

Only throat discomfort showed a significant improvement (*p * = 0.031) after surgery. On the other hand, swallowing and voice disorders persisted after surgery in 82.3% and 77.3% of patients, respectively (*p * = 0.250 and *p * = 0.062), such as the correlated reflux laryngopharyngitis (*p * = 0.5).

**Conclusions:**

LPR can be considered a predisposing factor or an important concurrent causa to the persistence of LNS after TT, in particular for swallowing disorders and voice disorders. In patients with non-toxic MNG who complain of local neck symptoms, the investigation of a possible coexistence of a reflux disease is appropriate before surgery. Patients should be informed about the possibility that some symptoms can persist even after removal of the goiter.

## Introduction

Non-toxic MNG affects 25–33% of the population in endemic areas [1] and LNS such as voice disorders, swallowing and throat discomfort are present in a range of 13–50% of these patients [2, 3]. However, the relationship between LNS and mechanical effects of MNG or its removal is not clear. In fact, swallowing disorders, voice disorders and throat discomfort may be related to goiter, but they are also reported by patients suffering from LPR [4, 5].

Reflux laryngopharyngitis is a fairly frequent finding caused by LPR, and is considered one of the atypical clinical presentations of gastroesophageal reflux affecting the larynx and pharynx, more often in the absence of gastrointestinal symptoms [6]. LPR prevalence is about 50% in patients with throat disorders [6, 7], it gives rise to swallowing and voice disorders and throat discomfort but catheter or capsule-based pH and impedance monitoring are unreliable to diagnose it [8, 9]. A highly significant correlation between symptoms and reflux laryngopharyngitis has been demonstrated [10].

However, patients who have undergone uncomplicated TT for MNG may experience symptoms such as swallowing disorders, hoarseness, a sensation of strangling or of a lump in the neck, a cough and sore throat, voice disorders, even long time after surgery. These symptoms have been related to a supposed injury of the perivisceral neural plexus that innervates the pharynx and larynx [2] or to a healing process that presumably resulted in laryngotracheal fixation with impairment of vertical movements [5, 11]. Swallowing, voice and throat discomfort should, therefore, be related in some way to the mechanical effects of MNG or TT, but in both of these cases the relationship between them and the thyroid mass or its removal is not always clear or easily demonstrable.

Our own previous study [4] found a correlation between MNG and LPR in select patients with LNS who had undergone uncomplicated TT, suggesting that the persistence of some LNS after surgery could be related to the coexistence of reflux laryngopharyngitis consequent to LPR.

The purpose of the present study was to investigate whether the coexistence of a LPR could play a role in the persistence of some LNS after uncomplicated TT in patients with MNG.

## Materials and methods

The study was performed on 212 consecutive patients with preoperative diagnosis of non-toxic MNG who underwent TT at our Institution in the period between January 2018 and December 2019. The indication for an elective TT was at all times discussed and established with the referring endocrinologist after an accurate preoperative workup. The main indications for TT were an increase in the size of the goiter during treatment with L-Thyroxine, an increase in the volume of one or more nodules even without treatment, the appearance of LNS. Exclusion criteria from the study included nicotine and/or alcohol abuse, previous head and neck surgeries and the patient’s refusal to be part of the study.

Each patient was investigated for both thyroid pathology and LPR. An accurate anamnesis, particularly focused on any smoke and/or alcohol abuse, the presence of allergies and previous head and neck surgeries, was conducted. The diagnostic preoperative workup included thyroid hormones, thyroid stimulating hormone (TSH) and antibodies assay, ultrasound examination of the thyroid and fine needle aspiration biopsy for cytological examination, if indicated. Moreover, video laryngoscopy with flexible optical fibers (VLS) and a video fluoroscopic swallowing study (VFSS) were performed.

At VLS, in addition to the evaluation of vocal cords motility, according to Reflux Finding Score (RFS) [12] the following findings were considered to assess the posterior laryngitis: erythema/hyperemia, vocal fold edema, diffuse laryngeal edema, thick endolaryngeal mucus, granuloma/granulation, pseudosulcus vocalis, ventricular obliteration, and posterior commissure hypertrophy. VLS was considered positive if at least two of the above findings were present.

At VFSS, the following parameters were considered for each patient to evaluate the swallowing alterations: hyoid elevation and epiglottic tilting; stasis of the bolus in the pharynx, valleculae and/or pyriform sinuses. VFSS was considered positive even if only one of the two conditions, laryngeal movement alterations or stasis of bolus, was present.

Cricopharyngeal contraction and gastroesophageal reflux (GER) in the middle/ upper esophagus provoked with a water siphon test (WST) were also detected.

All patients were interviewed by the same experienced clinician according to Reflux Symptom Index (RSI) [13]. For a more suitable evaluation for the purpose of the study LNS were subsequently classified into three different groups. This to include in the evaluation some symptoms reported by the patients but not included in the RSI and, moreover, to condense a fair number of symptoms into three single types of ailments. A first group made up of swallowing disorders or dysphagia, reported as a sensation of difficulty when swallowing liquids, solids and pills. A second group made up of voice disorders, reported as hoarseness or problems with the voice such as vocal fatigue or breaks and throat clearing. Inclusion in this group comprised the joint presence of both symptoms. A third group, consisting of throat discomfort, reported as excess throat mucus or post-nasal drip, coughing after meals or after lying down, breathing difficulties or choking episodes, troublesome or annoying cough and the sensation of something sticking in throat or a lump in throat. Inclusion in this third group comprised at least three of the five symptoms.

The assessment of symptoms before and after the operation was carried out considering the association between the three groups of symptoms. According to this criterion, the presence of only one of the three groups of symptoms, two of the three groups and all three groups of symptoms was considered for each patient. Both the interviews according to RSI and the VLS were performed before and 6 months after the surgery.

Out of the 212 patients, 32 were excluded for nicotinic and/or alcohol abuse, 20 refused to be part of the study. At the end, 160 patients were considered for the study. The patients’ characteristics are summarized in Table 1.

**Table 1:** Patients’ characteristics

Sex	
Male	44 (27.5%)
Female	116 (72.5%)
Age	47.3 years (range 16–77)
Histological diagnosis	
Non-toxic nodular goiter	104 (65%)
Non-toxic nodular goiter coexistent with Hashimoto’s thyroiditis	24 (15%)
Non-toxic nodular goiter coexistent with incidental differentiated thyroid carcinoma	32 (20%)
Tracheal deviation	21 (13.1%)
Tracheal compression	3 (1.9%)
WHO goiter grading [18]	
0	21 (13.1%)
1	65 (40.6%)
2	74 (46.2%)
Thyroid weight	
≤ 20 g	19 (11.9%)
20 to ≥ 40 g	63 (39.4%)
> 40 g	78 (48.7%)
Local symptoms	
Present	104 (65%)
Absent	56 (35%)

The surgical intervention consisted of total extracapsular thyroidectomy with systematic identification of recurrent laryngeal nerves and with low and selective ligature of the superior vascular peduncle to avoid injuries to the external branch of the superior laryngeal nerve. All surgical procedures were always performed by the same team of experienced surgeons, using intraoperative nerve monitoring to preserve the inferior laryngeal nerve and the external branch of the superior laryngeal nerve. No patients required a reoperation for bleeding and none suffered permanent hypoparathyroidism or recurrent palsy. Transient recurrent palsy developed in four (2.5%) patients. Symptomatic transient hypoparathyroidism, confirmed by low serum levels of calcium, was recorded in 36 (22.5%) patients and resolved with a calcium replacement within 8–12 weeks after surgery.

The Wilcoxon test was applied with a probability level equivalent to 95% to evaluate the difference in points obtained for each symptom through RSI and RFS before and after the operation. The results with a *p* value lower than 0.05 (*p *<0.05) are considered significant.

McNemar’s test was employed for the comparison of the occurrence of the groups of symptoms before and after surgery. Statistical evaluation was made considering either one single group of symptoms or two or three groups simultaneously.

Proportions and 95% Confidence Interval of positive tests in patients having one, two or three groups of symptoms were computed for VLS, before and after surgery. The statistical analysis was carried out using STATA software version 9.2.

This study was approved by the Ethical Committee of the University Hospital “Policlinico P. Giaccone”. Each patient was informed about the study in detail and gave their consent to participate.

## Results

Out of the 160 patients examined, 56 were asymptomatic for LNS, while 104 presented at least one group of symptoms before the operation.

In a first phase, statistical analysis was carried out on all 160 patients examining the sampling distribution of the 9 items of the RSI with the aim of measuring the intensity of the symptoms before and after the operation.

The median and average values for each symptom before and after surgery are reported in Table 2. A significant improvement after TT was observed for all the symptoms. In addition, none of the asymptomatic patients before surgery reported the appearance of symptoms after TT.

**Table 2:** Features of the 160 patients and comparison of RSI before and after surgery

RSI	Pre-surgery median (average)	Post-surgery median (average)	*p* value
Hoarseness or problem with the voice	1.0 (1.32)	0.0 (0.85)	0.0001
Throat clearing	1.5 (1.35)	0.0 (0.87)	0.0001
Excess throat mucus or post-nasal drip	0.5 (1.32)	0.0 (0.82)	0.0000
Difficulty swallowing food, liquids or pills	0.0 (1.05)	0.0 (0.77)	0.0013
Coughing after meals or after lying down	0.0 (0.65)	0.0 (0.45)	0.0047
Breathing difficulties or choking episodes	0.0 (0.25)	0.0 (0.25)	*
Troublesome or annoying cough	0.0 (0.90)	0.0 (0.65)	0.0082
Sensations of something sticking in throat or a lump in throat	1.0 (1.12)	0.0 (0.77)	0.0001
GER symptoms (heartburn, chest pain, indigestion and regurgitation)	1.0 (1.45)	0.0 (0.95)	0.0001
Total	4 (9.40)	1.5 (6.25)	0.0000

*The symptom has no difference before or after surgery. According to the Wilcoxon test no ranking is attributed and the correspondent statistical unit is null

Table 3 shows the number of patients positive to each symptom before surgery and, in the final column, it reports the number of patients in whom the same symptom disappeared or improved after surgery. Before surgery, the main symptoms were “GER symptoms” (62.5%) followed by “throat clearing” symptom (60%) and “hoarseness or voice problems” (57.5%). After the operation, the symptoms which saw a greatest reduction were “excessive throat mucus or post-nasal drip” (30%), “coughing after meals or lying down” (28.6%), “something sticking in the throat sensation” (28.6%).

**Table 3:** Improvement/disappearance of each symptom after surgery among the 160 patients

Symptoms	Symptomatic before surgery No. patients (%)	Improvement/disappearance of the symptom after surgery No. patients (%)
Hoarseness or problem with the voice	92 (57.5%)	20 (21.7%)
Throat clearing	96 (60%)	24 (25%)
Excess throat mucus or post-nasal drip	80 (50%)	24 (30%)
Difficulty swallowing food, liquids or pills	68 (42.5%)	12 (17.6%)
Coughing after meals or after lying down	56 (35%)	16 (28.6%)
Breathing difficulties or choking episodes	8 (5%)	0
Troublesome or annoying cough	52 (32.5%)	8 (15.4%)
Sensations of something sticking in throat or a lump in throat	84 (52.5%)	24 (28.6%)
GER symptoms (heartburn, chest pain, indigestion and regurgitation)	100 (62.5%)	24 (24%)

*n*_*p*_ patients positive to symptom, *N* − *n*_*p*_ patients negative to symptom

Considering that this result may have been influenced by the fact that 35% of the sample (56 patients) was already asymptomatic before thyroidectomy, in a second phase, only 104 patients presenting LNS before surgery were considered to appreciate which groups of symptoms persisted after the operation.

Table 4 shows the results obtained by comparing the presence of both individual symptoms and groups of symptoms before and after the operation using the McNemer test. Symptoms including “throat clearing”, “excessive throat mucus or post-nasal drip”, “something sticking in the throat symptom”, “GER symptoms” have shown a significant reduction after surgery. Postoperative analysis of symptom groups showed a significant reduction only for “throat discomfort” symptoms. Finally, a noticeable reduction of the symptoms was recorded after the operation exclusively in those patients who had all three groups of symptoms. No patient in the preoperative stage presented two groups of symptoms; however, after the operation 5 patients (4.8%) showed the appearance of two groups of symptoms. Finally, Table 4 shows how reflux pharyngolaryngitis persisted after the operation in most patients.

**Table 4:** Comparison of single symptoms and groups of symptoms before and after surgery in 104 symptomatic patients

	Before surgery No. of pts (%)	After surgery No. of pts (%)	*p* value
Symptoms			
Hoarseness or problem with the voice	92 (88.5)	72 (69.2)	0.062
Throat clearing	96 (92.3)	72 (69.2)	0.031
Excess throat mucus or post-nasal drip	80 (76.9)	56 (53.8)	0.031
Difficulty swallowing food, liquids or pills	68 (65.4)	56 (53.8)	0.250
Coughing after meals or after lying down	56 (53.8)	40 (38.5)	0.125
Breathing difficulties or choking episodes	8 (7.7)	8 (7.7)	1
Troublesome or annoying cough	52 (50)	44 (42.3)	0.500
Sensations of something sticking in throat or a lump in throat	84 (80.8)	60 (57.7)	0.031
GER symptoms (heartburn, chest pain, indigestion and regurgitation)	100 (96.1)	76 (73.1)	0.031
Groups of local neck symptoms			
Swallowing disorders	68 (65.4)	56 (53.8)	0.250
Voice disorders	88 (84.6)	68 (65.3)	0.062
Throat discomfort	64 (61.5)	40 (38.4)	0.031
Association of local neck symptoms groups			
One group of symptoms	28 (26.9)	20 (19.2)	0.500
Two groups of symptoms	–	20 (19.2)	1
Three groups of symptoms	64 (61.5)	36 (34.6)	0.015
VLS			
Reflux Laryngopharyngitis	80 (76.9)	72 (69.2)	0.500

Table 5 indicates the proportion and 95% CI of patients, considered on the basis of the association of the three groups of symptoms, in which the VLS was positive to LPR. Data show that LPR has not improved, or rather worsened, after surgery.

**Table 5:** Proportion of positive VLS e IC al 95% in symptomatic patients to three groups of symptoms

Local neck symptoms	VLS proportion (*n*) 95% CI		
	Before surgery	After surgery	*p* value two sample test proportion
One group	0.57 [0.18–0.90]	0.40 [0.05–0.85]	0.558
Two groups	–	0.20 [0.005–0.72]	–
Three groups	0.44 [0.20–0.70]	0.78 [0.40–0.98]	0.100

## Discussion

The persistence of LNS after a thyroidectomy for MNG has been the subject of considerable interest in the last decade. The same symptoms, in fact, are common in the general population and are often attributed to LPR and reduced by the treatment of this.

Pereira et al. [2] analyzed the question, highlighting the appearance of the so-called “subjective non-specific aero-digestive symptoms” after an uncomplicated thyroidectomy, attributing responsibility for these disorders to the surgical manipulation of the perithyroidal nerve plexus, which innervates the pharynx and larynx. Hence, he considered those symptoms as a consequence of the surgery. Many authors have reported the presence of such symptoms in patients undergoing thyroidectomy suggesting the same pathogenetic mechanism. Other authors have, instead, hypothesized the possible relationship between the persistence of some symptoms after thyroidectomy and the coexistence of other pathologies with the same clinical presentation. [11].

In 2011, in our previous study [4], we concluded that LPR should be considered as a possible cause of the persistence of some LNS after a thyroidectomy, since thyroidectomy alone has not proven to be a conclusive solution. The merit of the study was to highlight how an unknown or poorly managed reflux disease can coexist in some patients with MNG and how some LNS can be erroneously attributed to mechanical action (compression) produced by the goiter rather than reflux disease. The study showed that these symptoms persisted or sometimes increased even after surgery. The significant difference of this study, compared to others, was that it aimed to demonstrate that the symptoms were not caused by the surgery, but that they could be misunderstood before surgery and exacerbated by it. Our etiopathogenetic hypothesis was based on a careful preoperative and postoperative study of patients with LNS candidates for TT, investigating a possible correlation with LPR, which the study showed.

Hamdan et al. [14] recognized the merit of our study for being the first to examine such association, highlighting, however, that the enrollment of only patients complaining of LNS was a limit. Interestingly, they compared the results of our first study with their own. The persistence of dysphagia and dysphonia for more than 3 months after surgery was 79% in our study and 75% in them, respectively, confirming the importance of a careful preoperative study, not to mistakenly attribute these symptoms to a consequence of the surgical procedure. This evidence was confirmed in 2014 by the American Thyroid Association Statement on Optimal Surgery [15], which, in the new guidelines for goiter treatment, underlines the need to make a differential diagnosis between the two diseases through a careful study of the swallowing mechanics before the surgery.

Therefore, taking Hamdan’s observations into account, the idea of starting a second study arose, following the aim of the previous one, but on a larger sample, recruiting the patients candidates for TT regardless of the presence of LNS. The possible occurrence of LNS after TT in those patients who were asymptomatic before surgery would, for example, have questioned our initial hypothesis and the results of the first study, once again putting the role of surgery as cause of these disorders.

The present study substantially confirms the results of our previous work. Analyzing the data relating to the entire sample of 160 patients, a significant first finding was that none of the asymptomatic patients before TT complained of any symptoms after surgery, once again downsizing the role of surgery as cause of developed or worsened LNS. Furthermore, TT achieved a significant improvement in reducing all symptoms, particularly the “excessive throat mucus or post-nasal drip”, “coughing after meals or lying down” and “something sticking in the throat sensation”, all likely attributable to the compression exerted by the goiter. However, a bias against these findings could be that approximately 35% of the sample consisted of asymptomatic patients before surgery, who experienced no symptoms after surgery.

Instead, considering only the 104 patients who presented LNS before surgery, only the subgroup of patients who complained of “throat discomfort” and the one who presented all three groups of symptoms experienced a statistically significant postoperative improvement. This could be plausibly explained by the fact that the removal of the goiter contributes to the improvement of those symptoms most likely attributable to the mechanical compression exerted by the goiter itself.

The association of the two groups of symptoms, which were absent before surgery, appeared in 5 (4.8%) patients after TT. The data can be interpreted as the disappearance of throat discomfort in some patients who previously presented three groups of symptoms.

Finally, no significant improvement was recorded at postoperative VLS for the reflux larynopharyngitis. This finding is also confirmed by the fact that in patients presenting the association of the three groups of symptoms there was a significant increase in LPR after TT. Postoperative worsening of the reflux laryngopharyngitis, does not have a certain etiopathogenetic hypothesis yet. In our pervious article [16] we hypothesized that thyroidectomy may worsen the LPR by causing a malfunctioning of the upper esophageal sphincter and /or impairing the movements of hyoid–larynx elevation.

## Conclusions

The main limitation of this study could be the relatively low number of patients recruited, considering the high incidence of both multinodular goiter and gastroesophageal reflux in the general population. However, it has the merit of confirming that LPR can be considered a predisposing factor or an important concurrent causa to the persistence of some LNS after TT, in particular for swallowing and voice disorders. Therefore, in patients with non-toxic MNG who complain of local neck symptoms, the investigation of a possible coexistence of a reflux disease is appropriate before surgery, as also recommended by the American Thyroid Association Statement on Optimal Surgery. If this is present, patients should be well informed about the possibility that some symptoms can persist even after removal of the goiter. Furthermore, as suggested by some authors to minimize the persistence of swallowing and voice disorders [17], it could be recommended to treat these patients with proton pump inhibitors a few weeks before the intervention, continuing the treatment for at least 3 weeks after surgery.

## Data Availability

The data sets used and/or analyzed during the current study are available at the Department of Surgical Oncological and Oral Sciences, University of Palermo, Via del Vespro 129 90127 Palermo Italy and can reasonably requested to corresponding author.
